# Inhibition of ERK1/2 in cancer-associated pancreatic stellate cells suppresses cancer–stromal interaction and metastasis

**DOI:** 10.1186/s13046-019-1226-8

**Published:** 2019-05-27

**Authors:** Zilong Yan, Kenoki Ohuchida, Shuang Fei, Biao Zheng, Weiyu Guan, Haimin Feng, Shin Kibe, Yohei Ando, Kazuhiro Koikawa, Toshiya Abe, Chika Iwamoto, Koji Shindo, Taiki Moriyama, Kohei Nakata, Yoshihiro Miyasaka, Takao Ohtsuka, Kazuhiro Mizumoto, Makoto Hashizume, Masafumi Nakamura

**Affiliations:** 1 Department of Surgery and Oncology, Graduate School of Medical Sciences, Kyushu University, 3-1-1 Maidashi, Fukuoka, 812-8582 Japan; 2 Department of Advanced Medical Initiatives, Graduate School of Medical Sciences, Kyushu University, Fukuoka, Japan; 3 Department of General Surgery, Shenzhen University General Hospital, Shenzhen, China; 4 Cancer Center of Kyushu University Hospital, Fukuoka, Japan

**Keywords:** ERK1/2, Pancreatic cancer, Cancer–stromal interaction, Pancreatic stellate cell, Cellular senescence

## Abstract

**Background:**

Extracellular signal-regulated kinases (ERKs) have been related to multiple cancers, including breast cancer, hepatocellular cancer, lung cancer and colorectal cancer. ERK1/2 inhibitor can suppress growth of *KRAS*-mutant pancreatic tumors by targeting cancer cell. However, no studies have shown the expression of ERK1/2 on pancreatic stromal and its effect on pancreatic cancer–stromal interaction.

**Methods:**

Immunohistochemistry and western blotting were performed to detect the expression of p-ERK1/2 in pancreatic tissues and cells. Cell viability assay was used to study IC50 of ERK inhibitor on pancreatic cancer cells (PCCs) and primary cancer-associated pancreatic stellate cells (PSCs). Transwell migration, invasion, cell viability assay, senescence β-galactosidase staining were performed to determine the effect of ERK inhibitor on PCCs and PSCs in vitro and in vivo. The expression of key factors involved in autophagy and epithelial-to-mesenchymal transition (EMT) process were evaluated by western blotting. The expression of key factors related to cell invasiveness and malignancy were confirmed by qRT-PCR. Co-transplantation of PCC Organoid and PSC using a splenic xenograft mouse model was used to evaluated combined treatment of ERK inhibitor and autophagy inhibitor.

**Results:**

Immunohistochemical staining in pancreatic tumor samples and transgenetic mice detected p-ERK1/2 expression in both cancer cells and stromal cells. In pancreatic tissues, p-ERK1/2 was strongly expressed in cancer-associated PSCs compared with cancer cells and normal PSCs. PSCs were also significantly more sensitive to ERK1/2 inhibitor treatment. Inhibition of ERK1/2 suppressed EMT transition in HMPCCs, upregulated cellular senescence markers, activated autophagy in cancer-associated PSCs; and suppressed cancer–stromal interaction, which enhanced invasiveness and viability of cancer cells. We also found that chloroquine, an autophagy inhibitor, suppressed ERK inhibition-induced autophagy and promoted PSC cellular senescence, leading to significantly decreased cell proliferation. The combination of an ERK inhibitor and autophagy inhibitor suppressed liver metastasis in a splenic pancreatic cancer organoid xenograft mouse model.

**Conclusions:**

These data indicate that inhibition of ERK1/2 in cancer-associated pancreatic stellate cells suppresses cancer–stromal interaction and metastasis.

**Electronic supplementary material:**

The online version of this article (10.1186/s13046-019-1226-8) contains supplementary material, which is available to authorized users.

## Introduction

Pancreatic ductal adenocarcinoma (PDAC) is the fourth most common cause of cancer death worldwide, with a 5-year overall survival (OS) rate of only 5% [1]. The overwhelming majority of pancreatic cancer patients are diagnosed with liver metastasis [2], which is the leading primary cause of PDAC death, with a 5-year OS rate of 2.7% [3], and a median OS of less than 6 months [2]. As curative resection is not feasible after PDAC metastasizes to the liver [4], novel therapeutic strategies and agents are urgently needed in this setting.

Pancreatic cancer is characterized by excessive desmoplasia, which exhibited abundant tumor stromal in histology [5]. Tumor–stromal interactions reportedly promote PDAC progression and resistance to chemotherapies [6]. Pancreatic stellate cells (PSCs) are the primary stromal contributors to fibrosis in pancreatic disease [7]. PSCs transform from quiescent cells to activated myofibroblast-like cells through various stimuli, including interactions with tumor cells. Activated PSCs secrete cytokines that promote tumor cell proliferation and invasion [8]. As highly fibrotic stromal cells are seen in PDAC tumors and metastases, targeting stromal cells could be a therapeutic approach to PDAC [9].

Mitogen-activated protein kinases (MAPKs), also known as extracellular signal-regulated kinases (ERKs), act as an integration point for multiple biochemical signals, and affect such cellular processes such as proliferation, differentiation, transcription and development [10]. Two linked members of the MAPK family, ERK1 and ERK2, have been related to multiple human cancers, including breast cancer [11, 12], hepatocellular cancer [13], lung cancer [14] and colorectal cancer [15]. Two recent articles highlighted the functional role of p-ERK1/2 in pancreatic cancer and the therapeutic potential of inhibiting ERK1/2 activation. Principe et al. found p-ERK1/2 is necessary for TGFβ-induced epithelial–mesenchymal transition (EMT) in pancreatic cancer cells (PCCs), and found inhibition of p-ERK1/2 to both reduce CDK2 levels and prevent EMT [16]. Hayes et al. used ERK1/2 inhibitor on *KRAS*-mutant PDAC cells, and discovered an association between p-ERK1/2 and downstream c-MYC, with effects on cancer cell growth suppression and cellular senescence [17]. To date, however, no studies have shown the effects of ERK1/2 inhibitors on PSCs derived from pancreatic cancer tissues.

In the present study, we provide the first investigation of p-ERK1/2 expression on PSCs, and evaluate the sensitivity of PSCs to ERK1/2 inhibitor. We assessed the effects of ERK1/2 inhibitors on human PDAC cancer–stromal interaction. We also found that combining ERK1/2 inhibitor with chloroquine (CQ), an autophagy inhibitor, remarkably suppressed cancer–stromal interaction on cancer progression, both in vitro and in vivo. Taken together, our findings suggest that ERK1/2 promotes pancreatic cancer–stromal interaction and metastasis, and is a promising target for treatment of PDAC.

## Materials and methods

### Pancreatic tissues

We obtained PDAC specimens from patients who underwent pancreatectomy for at our institution. KPC (LSL-Kras G12D/+; LSL-Trp53R172H/+; Pdx-1-Cre) transgenic mice were described previous [18]. Tissues were embedded, sliced, stained and observed using an optical microscope (BZ-X710; Keyence, Osaka, Japan).

### Immunohistochemistry

We blocked endogenous peroxidase activity with methanol containing 0.3% hydrogen peroxidase. Antigen retrieval was performed by boiling samples in a microwave oven (citrate buffer, pH 6.0). Human pancreatic tissues and KPC mice tissues were sliced to 4 μm, incubated with rabbit anti-phospho-ERK1/2 (#4370, Cell Signaling, Technology, Danvers, MA, USA) overnight at 4 °C and stained with EnVisionþ System-HRP Labeled Polymer Anti-Rabbit (#K4003; Dako, Glostrup, Denmark). The staining was performed using serial sections.

### Cells and culture conditions, and treatment

We used the following PCC lines: AsPC-1, CFPAC-1(American Type Culture Collection, Manassas, VA, USA), Panc-1 (Riken BioResource Center, Ibaraki, Japan), SUIT-2 (Japan Health Science Research Resources Bank, Osaka, Japan), and BxPC-3 (National Kyushu Cancer Center, Fukuoka, Japan). All PCCs were maintained in DMEM (Sigma Chemical Co., St. Louis, MO, USA) supplemented with 10% FBS at 37 °C with humidified 90% air and 10% CO_2_. Human pancreatic ductal epithelial (HPDE) cells were obtained from Dr. M.-S. Tsao (University of Toronto, Canada) and maintained in HuMedia-KG2 medium (KK-2150S Kurabo, Osaka, Japan). We established human PSCs from fresh pancreatic cancer surgical specimens using the outgrowth method [19–21], as described in our previous reports. The isolated cells were confirmed to be PSCs by their spindle-shaped morphology, and immunofluorescence staining for αSMA, vimentin, CD90, glial fibrillary acidic protein, and nestin, but not CK19 [22, 23]. They were used within eight passages for each assay. Immortalization of PSCs was conducted as described previous [23]. All PSCs were maintained in DMEM (Sigma-Aldrich Co., Tokyo, Japan) supplemented with 10% fetal bovine serum, streptomycin (100 mg/ml), and penicillin (100 mg/ml) at 37 °C in a humidified atmosphere containing 10% CO_2_. HPaSteC cells (#3830; ScienCell Research Laboratories, Carlsbad, CA, USA) were maintained according to the manufacturer’s instructions using Stellate Cell Medium (#5301; ScienCell). PCCs from primary tumors in KPC mice were established using an outgrowth method [19], and isolated cancer cell lines were maintained as described [24]. ERK1/2 inhibitor used in vitro (S7101, Selleck Chemicals, Houston, TX, USA) and in vivo (HY-50846, MCE, NJ, USA) were reconstituted following the manufacturer’s recommendations and used at the indicated doses. Chloroquine phosphate was purchased from Sigma-Aldrich (#PHR1258), dissolved in phosphate-buffered saline to 10 mM, and stored at ^−^20C until used.

### PDAC organoid culture

PDAC organoids were established from KPC PCCs, which were established using the outgrowth method as described [21]. PCCs were embedded in growth factor-reduced Matrigel (Cat#356231; BD Bioscience, CA, USA), and cultured in human complete medium at 37 C° for 14 days [21, 25]. Human complete medium was AdDMEM/F12 (Cat#12634–010; Invitrogen, CA, USA), medium supplemented with 1 M HEPES (Invitrogen), GlutaMax (Cat#35050–061; Invitrogen), penicillin/streptomycin (Cat#15140122; Invitrogen), B27 (Cat#17504044; Invitrogen), N-acetyl-l-cysteine (Cat#A9165; Sigma-Aldrich Co.), Wnt-3a (Cat#5036-WN-010; R&D Systems, MN, USA), R-Spondin 1 (Cat#120–38; Peprotech, NJ, USA), Noggin (Cat#120-10C; Invitrogen), epidermal growth factor (EGF, Cat#AF-100-15; Peprotech), fibroblast growth factor (FGF, Cat#100–26; Peprotech), nicotinamide (Cat#N0636; Sigma-Aldrich Co.), Y-27263 (Cat# Y0503; Sigma-Aldrich Co.) and A83–01 (Cat#2939/10; R&D Systems).

### Quantitative reverse transcriptional-polymerase chain reaction (qRT-PCR)

Total RNA was extracted from cultured cells using a High Pure RNA Isolation Kit (Roche Diagnostics, Mannheim, Germany) and DNase I (Roche Diagnostics, Sigma-Aldrich), according to the manufacturers’ instructions. We performed qRT-PCR using a QuantiTect SYBR Green Reverse Transcription-PCR kit (Qiagen, Tokyo, Japan) and a CFX96 Touch Real-Time PCR Detection System (Bio-Rad Laboratories, Hercules, CA). Our specific primer sequences were purchased from Sigma-Aldrich (Tokyo, Japan). Primer sequences are listed in Additional file 5: Table S1. We normalized mRNA expression levels to 18S rRNA levels.

### Matrigel invasion and migration assay

The invasiveness and migration capacities of PCCs were assessed by determining the number of cells invading or migrating across transwell chambers as previously described [24, 26]. For invasion assays, PCCs (1 × 10^5^ cells/250 μl) were seeded in the upper transwell chamber (8-μm pore size; Becton Dickinson, Franklin Lakes, NJ) containing 100 mL of reconstituted Matrigel-coated membrane (20 mg/well, BD Biosciences, Bedford, MA). Thereafter, cells were incubated for 36–48 h and the number of invading PCCs was counted. Cell migration assays were performed with PCCs using the same protocol as the invasion assay without a Matrigel-coated membrane. Cells were allowed to migrate, and were counted 18–24 h after cell seeding into the upper chamber. In co-culture experiments, PSCs were seeded in 24-well plates (#353504; Corning) at a density of 5 × 10^4^ cells/well. At 24 h after seeding, medium was replaced with 750 μL of fresh DMEM containing 10% FBS. Transwell chambers (8-μm pores; Becton Dickinson) were placed in 24-well dishes, and then PCCs, which had been suspended in 250 μL of the same medium (1 × 10^5^ cells/mL) were seeded into the transwell chambers. After incubation for the indicated time, migration and invasion were evaluated by counting the cells that had invaded to the lower chamber. In both assays and at each time point, invaded or migrated cells at the bottom of the chamber were fixed with 70% ethanol, stained with hematoxylin and eosin, and counted in 5 random fields at 100× magnification (BZ-X710; Keyence Corporation, Osaka, Japan). Each experiment was performed in triplicate and repeated at least three times.

### Western blot analysis

Western blotting was performed as described previously [27]. Cells were lysed in Pro-Prep (InTron Biotechnology, Seongnam, Korea) and proteins were separated on 4–15% Mini-Protean TGX Precast Gels (Bio-Rad) and transferred to Trans-Blot Turbo Mini PVDF Transfer Packs (Bio-Rad) using a Trans-Blot Turbo Transfer Starter System (Bio-Rad). Membranes were incubated overnight at 4–8 °C with anti-ERK1/2 (#4695, Cell Signaling Technology, Danvers, MA, USA), anti-phospho-ERK1/2 (#4370, Cell Signaling Technology), anti-αSMA (#M0851; Dako, Japan), anti-E-cadherin (#3195S, Cell Signaling Technology), anti-vimentin (#5741, Cell Signaling Technology), anti-collagen type I (sc-8783 Santa Cruz Biotechnology, Santa Cruz, CA, USA), anti-collagen type VI (sc-47,712, Santa Cruz Biotechnology), anti-MMP2 (#13132, Cell Signaling Technology), anti-MMP3 (#sc-6839, Santa Cruz Biotechnology), anti-MMP14 (AB8345, Millipore, Temecula, CA, USA), anti-IL-6 (#ab9324, Abcam, Cambridge, MA, USA), anti-p16 (MABE1328, Millipore), anti-p15 (MABE1664, Millipore), anti-CC8 (#9496S, Cell Signaling Technology), anti-fibronectin (sc6952, Santa Cruz Biotechnology), anti-LC3 (#2775S; Cell Signaling Technology), anti-AKT (#4060S, Cell Signaling Technology), anti-phospho-AKT (#4691S, Cell Signaling Technology), and anti-b-actin (ab8227; Abcam), and then probed with appropriate secondary antibodies (Cell Signaling Technology). Immunoblot signals were detected by enhanced chemiluminescence with ChemiDoc XRS (Bio-Rad).

### Cell viability assay

Cells (1 × 10^3^ cells/well) were seeded in 96-well plates (Greiner Bio-One, Frickenhausen, Germany) and cell viability examined using the CellTiter-Glo Luminescent Cell Viability Assay Kit (G7570, Promega) following the manufacturer’s instructions. Background was subtracted using values from wells containing only culture medium.

### In vivo experiments

BALB/c AJcl *nu/nu* female mice were purchased from Clea (Tokyo, Japan) and transported to our institution at 4 weeks old. After 1 week of acclimation, each of the 20 nude mice were splenic implanted with 5 × 10^4^ PDAC organoids and 5 × 10^4^ PSCs and randomized into four groups for treatment with PBS (control), ERK1/2 inhibitor (25 mg/kg dissolved in 100 μL PBS) alone, CQ (50 mg/kg dissolved in 100 μL PBS) alone, or with a combination of ERK1/2 inhibitor and CQ. One week after implantation, mice were treated intraperitoneally with either vehicle, SCH772984, Chloroquine or combination according to the dosing schedule indicated in the figure legends. The mouse liver metastasis tissues were fixed in formaldehyde, embedded in paraffin, and cut into 4-μm-thick sections. All mouse experiments were approved by the Ethics Committee of Kyushu University.

### Statistical analysis

For in vitro experiments, values are expressed as mean ± standard deviation. Comparisons between two groups were made using Student’s *t*-test. *P* < 0.05 was considered significant.

## Results

### Establishment and characterization of highly metastatic PCCs

**Fig. 1:**
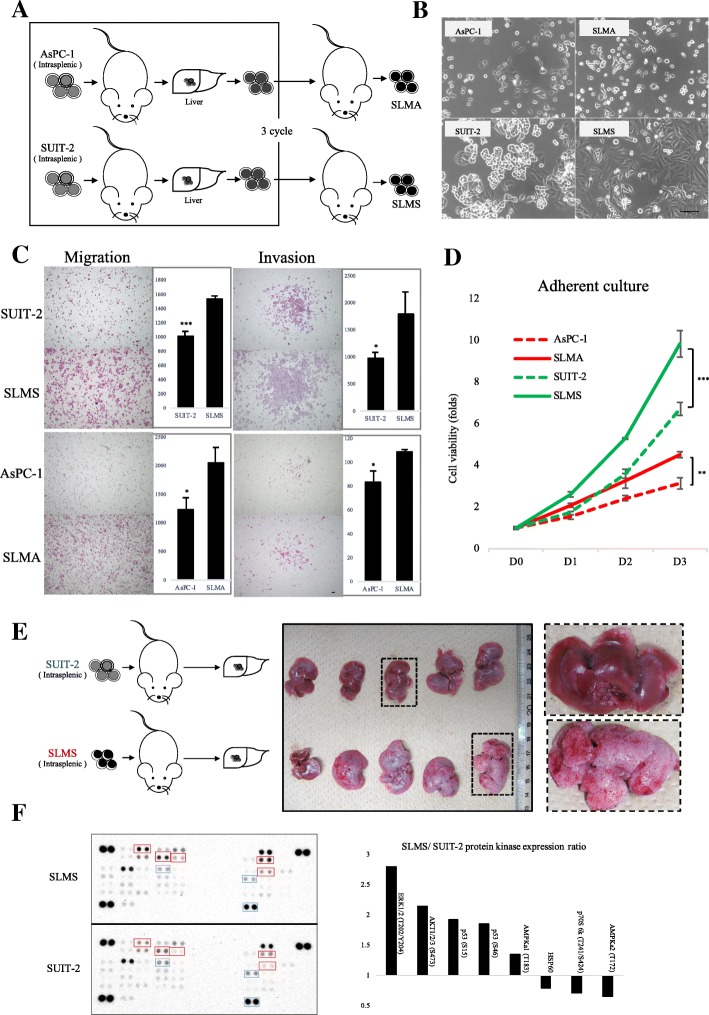
Establishment of highly metastatic PCCs. **a** Establishing PDAC cells. Parental PDAC cells were splenic transplanted into nude mice; liver metastases were harvested after 2–4 weeks. This process was performed 3 times. **b** Cellular morphology of parental PDAC cells and highly metastatic PDAC cells. Scale bars = 100 μm. **c** Migration and invasion assays were performed over 36 and 18 h respectively. Graphs show numbers of cells calculated from five fields. Original magnification: 40×. Scale bars =100 μm. *P < 0.05, ***P < 0.001. **d** Cell viability of cancer cells as determined by CellTiter-Glo luminescent cell viability assay. *P < 0.05, **P < 0.01. **e** SUIT-2 and SLMS cells were intrasplenic injected in nude mice and the liver metastases were harvested. Gross pathology indicated metastatic lesions. **f** Phospho-protein kinase array of SUIT-2 and SLMS cells. Right: most significant gene alterations

Three consecutive rounds of in vivo selection were performed by using splenic xenografts of PDAC cell lines SUIT-2 and AsPC-1 cells, from which metastatic lesions were harvested to establish metastatic SUIT-2 (SLMS) and AsPC-1 (SLMA) cells (Fig. 1a). We then investigated in vitro characteristics of the SLMS and SLMA cells. The SLMS cells had an apparent spindle-shaped morphology compared with their parental SUIT-2 cells (Fig. 1b). Migration, invasion and proliferation capacities of highly metastatic (HM) PCCs were significantly greater than their parental PCCs (Fig. 1c, d; Additional file 1: Figure S1A). We confirmed that SLMS cells occurred in liver metastases more frequently than did their parental SUIT-2 cells in vivo (Fig. 1e; Additional file 1: Figure S1B). As we had verified the upregulated aggressiveness of metastatic cells, we compared results of phospho-kinase array analysis between SLMS cells and parental SUIT-2 cells, and found altered expression of several phosphorylation kinases HMPCCs, including upregulation of p53 (S15), p53 (S46), AKT (S473), ERK1/2 (T202/Y204), AMPKa1 (T183), and downregulation of p70S6 kinase (T241/S424) and AMPKa2 (T172) (Additional file 1: Figure S1C). The greatest increase in phosphorylation level was seen in ERK1/2 (T202/Y204). To validate the accuracy of array data, we used western blotting to evaluate expression of p-ERK1/2 in HMPCCs and parental cells. We also found the mesenchymal marker vimentin was upregulated in HMPCCs (Additional file 1: Figure S1D). These data indicated that established HMPCCs were more aggressive and tumorigenic than their parent cells.

### Expression of p-ERK1/2 in pancreatic cancer tissues and cells

**Fig. 2:**
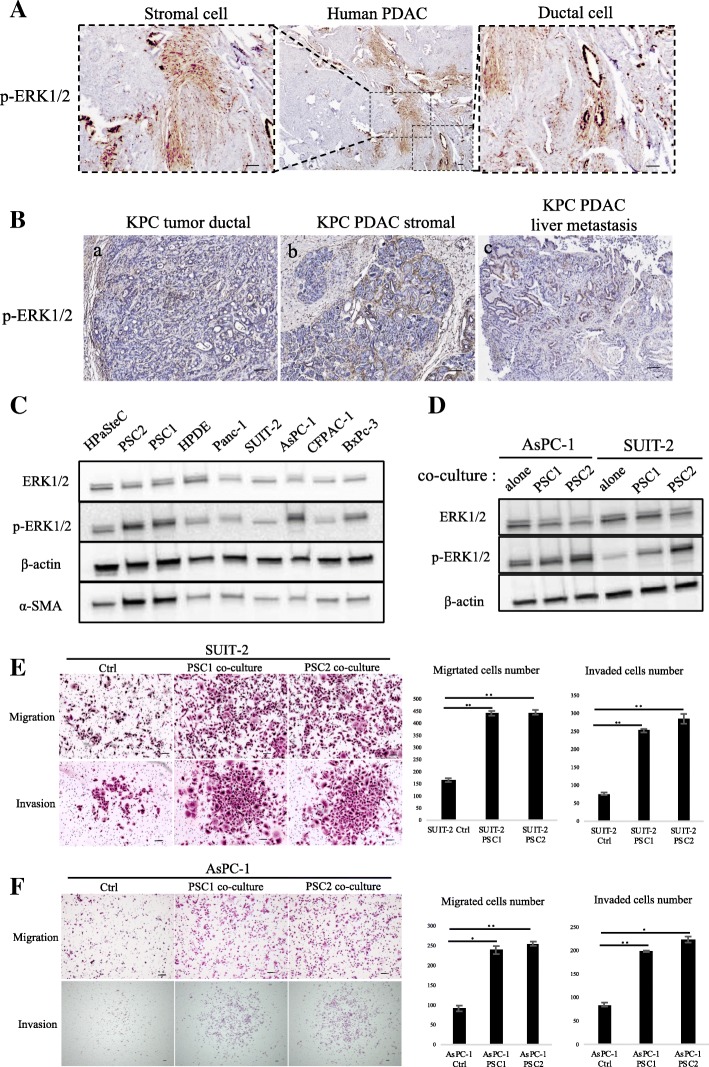
Expression of p-ERK1/2 in PDAC tissues and pancreatic cell lines. **a** p-ERK1/2 expression was detected in both pancreatic cancer cells and stromal cells. Scale bars =100 μm. **b** p-ERK1/2 expression was detected in KPC mice cancer cells (**a**) and stromal cells (**b**) of pancreatic primary tumor, and liver metastases (**c**). Scale bars =100 μm. **c** Western blot of ERK1/2, p-ERK1/2, and α-SMA levels in pancreatic cells. **d** Western blot of ERK1/2 and p-ERK1/2 levels in PCCs, alone or after co-culture with PSCs. PCCs were seeded in 24-well plates while PSCs were seeded in the upper transwell chamber with 3-μm pore size. **e** SUIT-2, (**f**) AsPC-1 Migration and invasion assays were performed for 18 and 36 h, respectively. PSCs were seeded in 24-well plates while PCCs were seeded in the upper transwell chamber of 8-μm pore size. Graphs show numbers of cells calculated from five fields. Scale bars =100 μm. *P < 0.05, **P < 0.01

In previous reports, p-ERK1/2 expression in PCCs was demonstrated in pancreatic cancer tissues. Using database of Human Protein Atlas (available from www.protenatlas.org), we found expression of ERK1 and/or ERK2 in both tumor and stromal cells (Additional file 2: Figure S2A, B). However, analysis of The Cancer Genome Atlas [28, 29] shows expression of ERK2, but not ERK1, correlates with poorer overall and disease-free survival in PDAC (Additional file 2: Figure S2C, D). We therefore investigated p-ERK1/2 expression in PDAC samples obtained in our institution (Additional file 6: Table S2) and detected p-ERK1/2 on both tumor cells and stromal cells (Fig. 2a). We also assessed p-ERK1/2 expression in PDAC derived from our KPC mouse model (LSL-KrasG12D/+; LSL-Trp53R172H/+; Pdx-1-Cre) and found p-ERK1/2 expression in primary tumor cells, stromal cells and liver metastases (Fig. 2b). Next, we investigated the p-ERK1/2 expression of various pancreatic cells including HPaSteC cells (normal PSC cells), cancer-associated PSCs, HPDE cells and PCCs. PSCs showed high p-ERK1/2 expression, even compared with PCCs (Fig. 2c). Interaction between PCC and PSC is a key process in pancreatic cancer progression [30]. To validate the involvement of p-ERK1/2, we co-cultured PCCs and PSCs, using the transwell system. Compared with monocultured cells, p-ERK1/2 expression was significantly upregulated in PCCs when co-cultured with PSCs (Fig. 2d). Previous study showed that PSCs can promote migration, invasion and EMT process via the regulation of E-cadherin and vimentin expression in PDAC cells [31]. We also found that cell migration and invasion of AsPC-1 and SUIT-2 were enhanced when indirectly co-cultured with PSCs (Fig. 2e and f).

### SCH772894 suppressed PCCs proliferation and epithelial–mesenchymal transition

**Fig. 3:**
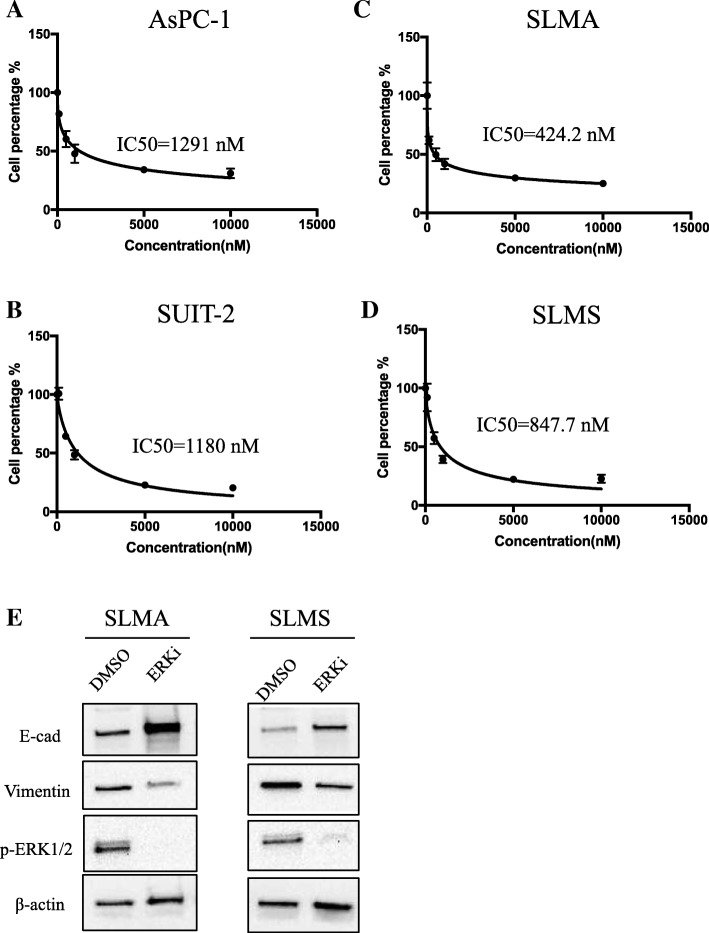
Inhibition of ERK1/2 decreased PDAC cell viability and EMT transition. **a** AsPC-1, (**b**) SUIT-2, (**c**) SLMA, and (**d**) SLMS cell viability after 72 h; treatment with various concentrations of ERK inhibitor after. IC_50_ values are indicated. **e** Western blot of E-cadherin, vimentin, and p-ERK1/2 levels of highly metastatic cancer cells after treatment with ERK inhibitor SCH772984 at IC_50_ value. The indicated protein was extracted exclusively from the living adherent cells. Negative control: DMSO

Next, we investigated the effect of ERK inhibition on PCCs and PSCs. We chose a novel ERK1/2-selective pharmacologic inhibitor, SCH772984, which has shown cellular potency in tumor cells with *BRAF*, *NRAS*, or *KRAS* mutations and induces tumor regressions in xenograft models at toxicity-free doses [32]. First, we examined the effects of SCH772984 on viability of parental PCCs and HMPCCs. The IC_50_ values of SCH772984 on AsPC-1 and SUIT-2 cells were 1291 nM and 1180 nM, respectively (Fig. 3a, b), compared with 424.2 nM and 847.7 nM, respectively, for HM SLMA and SLMS cells, which indicates HMPCCs are more sensitive to ERK1/2 inhibitor (Fig. 3c, d). As expression of p-ERK1/2 in PDAC is reportedly related to EMT [33], we investigated changes in kinase phosphorylation in HMPCCs after ERK1/2 inhibition. Upregulation of the epithelial cell marker, E-cadherin, and downregulation of the mesenchymal marker, vimentin, were observed through western blotting (Fig. 3e), which indicates that inhibiting p-ERK1/2 leads to suppression of EMT in HMPCCs.

### SCH772984 suppressed pancreatic stellate cell proliferation and induced upregulation of cellular senescence marker

**Fig. 4:**
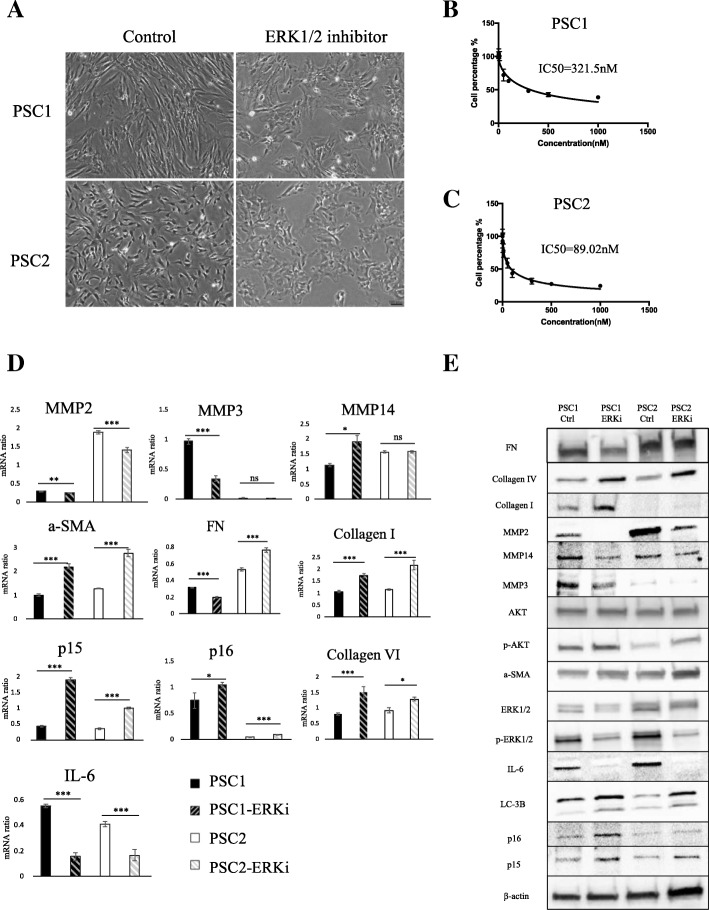
Inhibition of ERK1/2 facilitated PSCs atrophy and induces p16, α-SMA. **a** Microphotograph of PSCs after treatment with DMSO and/or ERK inhibitor. Scale bars = 100 μm. **b** Viability of PSC1 and (**c**) PSC2 cells, as determined by CellTiter-Glo luminescent cell viability assay after 72 h’ treatment with indicated concentrations of ERK inhibitor; IC_50_ values are indicated. **d** qRT-PCR of PSCs shows mRNA expression changes after ERK inhibitor treatment. *P < 0.05, **P < 0.01, ***P < 0.001. **e** The indicated protein levels of PSCs were evaluated after treatment with DMSO as control and/or ERK inhibitor SCH772984 at IC50 value

As high expression of p-ERK1/2 was only detected in PSCs (Fig. 2c), we hypothesized inhibiting ERK1/2 in PSCs would be more efficient than in PCCs. We established immortalized PSCs from a pancreatic cancer specimen obtained at our institution [34]. We observed a change from spindle-like shapes to round shapes among these PSCs after 72 h of SCH772984 treatment (Fig. 4a). The two primary cultures of PSCs were more sensitive to SCH772984, with IC_50_ values of 321 nM and 89 nM, respectively, compared with the HMPCCs (Fig. 4b, c). When we investigated changes in expressions of related cytokines and chemokines after SCH772984 treatment, we found senescence marker p15, p16, fibrosis marker α-SMA, fibronectin, Collagen Type I and Collagen Type IV were upregulated; and MMP2, MMP3, IL-6 (which are related to cell invasiveness and malignancy) were downregulated (Fig. 4d, e). These data are consistent with the results of the previous study, which showed that p16 induces cellular senescence and stable growth arrest without a senescence-associated secretory phenotype [35]. As inhibition of CDK4/6, a downstream target of ERK1/2, reportedly upregulated drug-induced autophagy in breast cancer [36], we investigated the effect of ERK inhibition on autophagy in PSCs. We found that autophagy marker LC-3II protein expression was upregulated. Our results suggest that inhibition of ERK did not induce the reversion of PSC from activated phenotype to quiescent type, but to cellular senescence, which may be another activated phenotype.

### SCH772984 suppressed cancer–stromal interactions in PCCs that enhance migration, invasiveness and viability

**Fig. 5:**
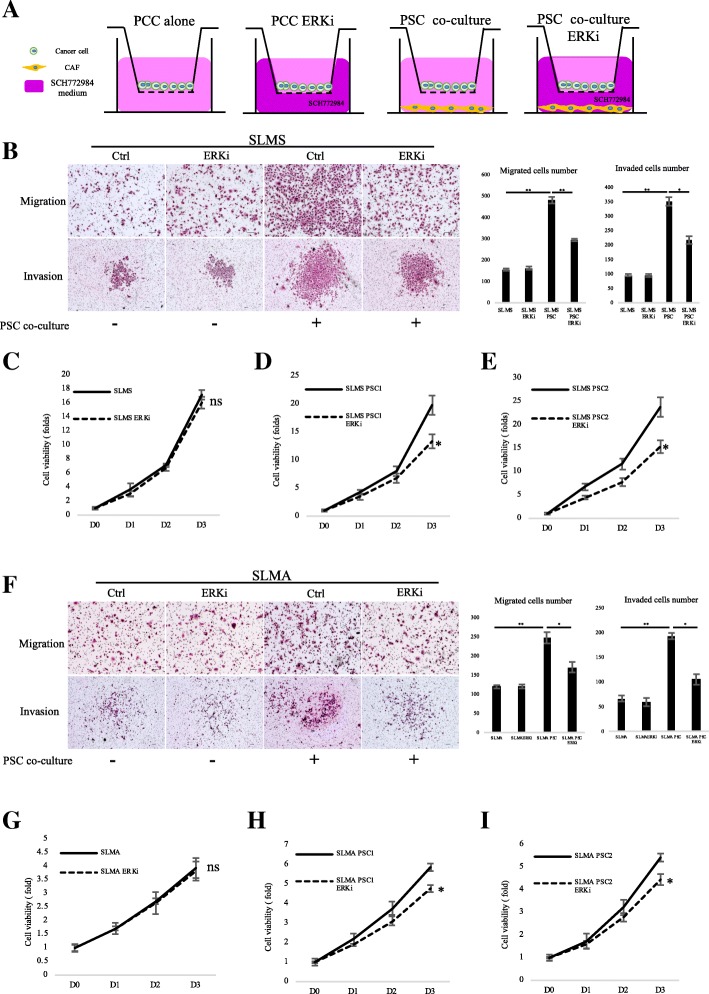
Inhibition of ERK1/2 suppressed PCC-PSC interaction by preferentially targeting PSC. **a** In indirect co-culture experiments, first PSCs were seeded, and 24 h later, medium was replaced and transwell chambers (8-μm pores; Becton Dickinson) were placed in 24-well dishes, and then PCCs were seeded into the transwell chambers. After incubation for the indicated time, migration and invasion were evaluated by counting the cells that had invaded to the lower chamber. SCH772984 dose was used IC50 value of PSC2 cells, 89 nM. **b** Migration and invasion assays were performed for 18 and 36 h, respectively. Graphs show numbers of cells calculated from five fields. Scale bars =100 μm. *P < 0.05, **P < 0.01. **c** Viability of SLMS cells co-cultured with (**d**) PSC1 or (**e**) PSC2 cells after DMSO or SCH772984 treatment; (**g**) Viability of SLMA cells co-cultured with (**h**) PSC1 or (**i**) PSC2 cells after DMSO or SCH772984 treatment was determined by CellTiter-Glo luminescent cell viability assay. SCH772984 dose was used IC50 value of PSC1, 321 nM; and PSC2, 90 nM. *P < 0.05. Columns, mean fold changes of three experiments done in triplicate

Because PCCs and PSCs showed similar reactions to SCH772984, we investigated the effect of SCH772984 on cancer–stromal interaction. Using a transwell indirect co-culture system (Fig. 5a), we found SCH772984 did not reduce PCC migration or invasion capacity when treated with the lower IC50 dose for PSCs. However, this lower dose of SCH772984 inhibited PCC migration and invasion when co-cultured with PSCs (Fig. 5b, f). In addition, a direct co-culture cell viability assay revealed SCH772984 suppressed proliferation of PCCs and PSCs co-cultured at the lower PSC IC50 dose (Fig. 5c-e and g-i). However, we did not obtain a similar finding for monocultured PCCs. These results suggest that treatment with SCH772984 could preferentially target PSCs to suppress cancer–stromal interaction.

### Combining SCH772984 with CQ suppressed fibrosis and induced senescence in PSCs

**Fig. 6:**
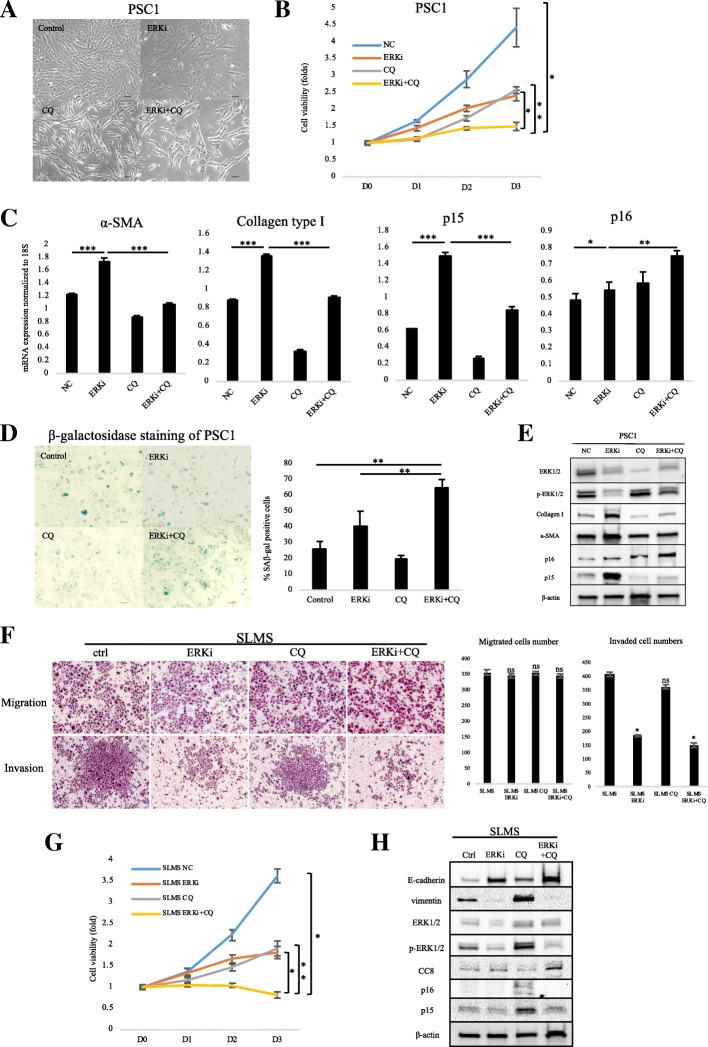
Dual treatment of SCH772984 and CQ decreased cell viability and induced senescence of PSCs. **a** Western blot of fibronectin, α-SMA, LC3-II, Akt and p-Akt levels of PSCs after treatment with ERK inhibitor. **b** Microphotograph of PSCs after indicated agent treatment. Scale bars = 100 μm. **c** Cell viability of PSCs after indicated agent treatment. **d** qRT-PCR shows mRNA expression of *α-SMA*, Collagen Type I, *p15* and *p16*. *P < 0.05, **P < 0.01, ***P < 0.001. **e** β-galactosidase staining of PSCs after indicated agent treatment. Bottom: graphs show the quantification of β-gal-positive cells calculated from five fields. Scale bars =100 μm. *P < 0.05. **e** Migration and invasion assays were performed for 18 and 36 h, respectively. Graphs show numbers of cells calculated from five fields. Scale bars =100 μm. *P < 0.05. **f** Cell viability of PCCs after indicated agent treatment. Columns, mean fold changes in three experiments done in triplicate. **g** Western blot of indicated protein levels in PCCs after indicated treatment

To evaluate therapeutic efficiency, we combined SCH772984 with an effective autophagy inhibitor CQ, which is shown to suppress PSCs activation via inhibition of autophagy activity [23]. A further morphology change was observed in PSCs after the combined treatment (Fig. 6a; Additional file 3: Figure S3a). Compared with a 4-fold increase in viability of control PSCs, the combined treatment remarkably restricted proliferation of PSCs (Fig. 6b; Additional file 3: Figure S3b); It also downregulated α-SMA and Collagen Type I expression, and upregulated senescence markers p15 and p16, compared with SCH772984 treatment alone (Fig. 6c; Additional file 3: Figure S3c). Primary cultured PSCs partly exhibited positive beta-galactosidase staining as shown in the previous reports [37]. We performed senescence β-galactosidase staining to investigate the correlation between p15/p16 expression and autophagy during PSC cellular senescence. We found SCH772984 treatment did not increase cellular senescence in PSCs, possibly due to autophagy activation, although p15/p16 expression was upregulated, and combined SCH772984 + CQ remarkably induced PSC cellular senescence (Fig. 6d; Additional file 3: Figure S3d). Therefore, combining SCH772984 with CQ suppressed drug-induced autophagy of SCH772984 and led to cellular senescence in PSCs.

### Combining SCH772984 with CQ suppressed cell viability and induced apoptosis in PCCs

Next, we evaluated the therapeutic effect of combine treatment of SCH772984 with CQ in PCCs. Single agent and/or combine treatment of SCH772984 and CQ didn’t affect the migration capacity of PCCs. SCH772984, but not CQ, significantly suppressed invasion of PCCs. And this effect was not further promoted by combine treatment with CQ. However, this effect was not further promoted by combine treatment with CQ (Fig. 6e; Additional file 3: Figure S3e). In addition, cell viability of PCCs was significantly suppressed by single agent of ERK inhibitor or CQ, and combination treatment further suppressed cell viability compared to single agent (Fig. 6f; Additional file 3: Figure S3f). Furthermore, the EMT transition was downregulated of PCCs following SCH772984 alone treatment and/or combination treatment. PCCs didn’t upregulated the cellular senescence markers p15 and p16 unlike PSCs. Instead, we observed an induction of CC8 expression, indicating combination treatment induced significant apoptosis of PCCs (Fig. 6g; Additional file 3: Figure S3g).

### Combination of SCH772984 and CQ inhibited metastases in KPC cancer organoid xenograft mouse model

**Fig. 7:**
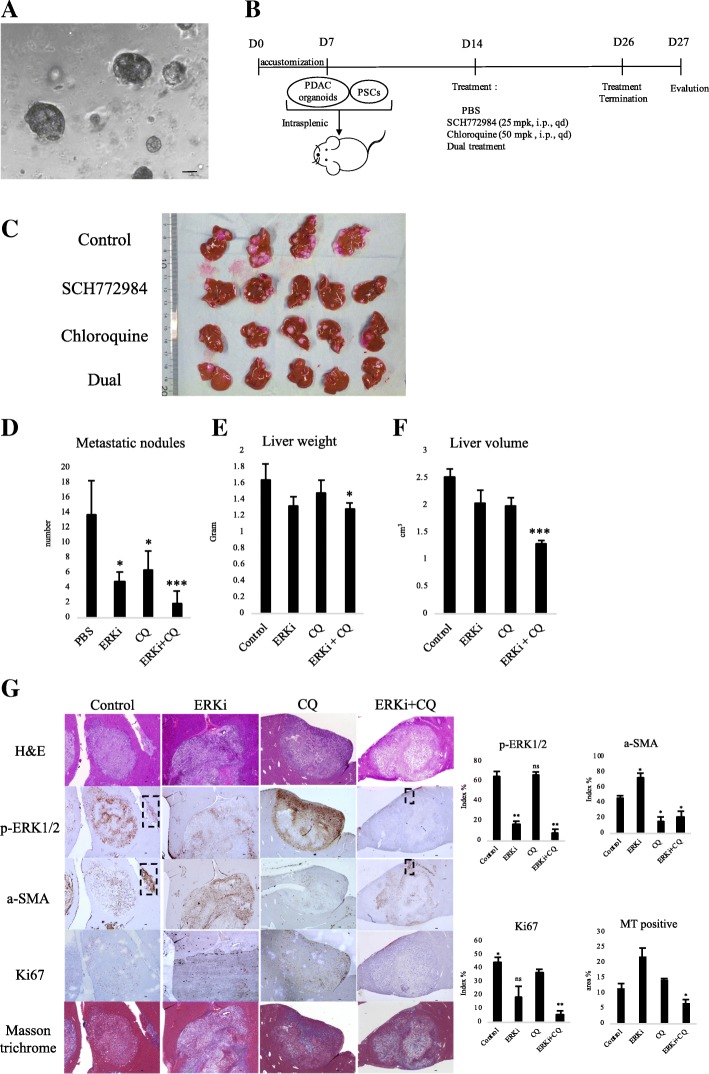
Dual treatment of SCH772984 and CQ decreased liver metastasis in xenograft organoid model with PSC co-transplantation. **a** Microphotograph of KPC mouse-derived cancer organoid. Scale bars =100 μm. **b** Scheme of xenograft experiment. Female nude mice were intrasplenic transplanted with cancer organoids with PSCs and randomized divided into four groups (*n* = 5/group). One week after implantation, mice were dosed once daily with vehicle, SCH772984 (25 mg/kg), Chloroquine (50 mg/kg), or dual treatment of each group for 13 days. Dosing occurred from day 14 to day 26. At day 27, mice were sacrificed and liver metastases were harvested. **c** Gross pathology showed significantly reduced liver metastasis formation after dual treatment of SCH772984 with chloroquine. **d** Liver metastasis nodules were significantly reduced in samples treated with SCH772984 or CQ, or both. *P < 0.05, ***P < 0.001. **e** Tumor weight and (**f**) volume were significantly decreased only in the dual-treatment group. *P < 0.05, ***P < 0.001. **g** Immunohistochemical staining shows decreased p-ERK1/2 expression in SCH772984 group, and decreased α-SMA expression in CQ group; The dual-treated group showed significant reductions of p-ERK1/2, α-SMA, Ki67 and collagen fibers. Corresponding rectangles indicated a-SMA-positive PSCs. Right: quantification of gene expression from five fields. Scale bars =100 μm. *P < 0.05, **P < 0.01

ERK1/2 inhibitor was identified as a potential therapeutic agent for primary liver cancer in organoid xenograft experiments [38]. A pancreatic tumor organoid was shown to recapitulate the histology and gene expression of its parental tumor [25, 39]. We therefore generated cancer organoids for 2 weeks using KPC-derived PDAC cells as described previous (Fig. 7a) [21]. Organoid or 2D-cultured cells were then splenic transplanted into nude mice with KPC-derived PSCs. Consistent with previous reports [17, 32], SCH772984 treatment did not cause any toxic effects such as body weight loss. Compared to 2D-cultured cells, there was no significant increase of liver metastasis in splenic xenograft experiment using organoid. However, the implanted tumor derived from organoids frequently restored high p-ERK1/2 expression, which was consistent with the original tumor (Additional file 4: Figure S4). Because of these results, we chose organoid xenografts for the following experiments. KPC cancer organoids were splenic co-transplanted into nude mice with KPC-derived PSCs. One week after implantation, mice were treated intraperitoneally with either vehicle, SCH772984, CQ or combination according to the dosing schedule indicated in the figure legends (Fig. 7b). After 13 days of treatment, their liver metastases were harvested and evaluated (Fig. 7c). Compared with controls, the combined treatment group remarkably decreased the metastatic nodules (14 vs. 2, average) in the liver (Fig. 7d), liver volume (2.53 vs. 1.29cm^3^, average) and liver weight (1.65 g vs. 1.29 g, average), although SCH772984 alone and CQ alone also decreased metastatic nodules (14 vs. 5, 14 vs. 6, average) (Fig. 7e, f). Histologic analysis by using serial sections demonstrated that Ki67 expression was downregulated in the combined treatment (6% positive) group compared with control (44% positive) group and/or single-agent treatment (18% positive for SCH772984 and 36% positive for CQ) groups (Fig. 7g). The corresponding rectangles indicated reduction of p-ERK1/2 expression in a-SMA-positive PSCs compared with controls. In addition, masson’s trichrome stain demonstrated significant decrease in the expression of collagen fibers (7% positive) in combined treatment group compared with control (12% positive) group and/or single-agent treatment (22% positive for SCH772984 and 14% positive for CQ) groups (Fig. 7g).

## Discussion

**Fig. 8:**
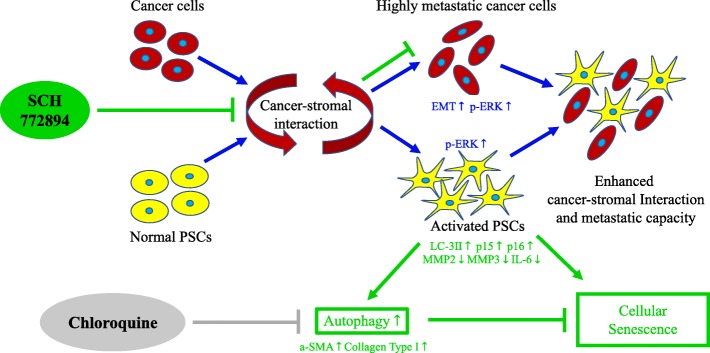
Mechanism of p-ERK1/2 inhibition on cancer–stromal interaction. In PDAC progression, interaction between cancer cells and stromal cells is a key regulator of ERK1/2 activation, during which cancer cells transform to an ERK1/2 activation phenotype and exhibit EMT transition tendency, and PSCs turn activated from their quiescent status. Thus enhancement of cancer–stromal interactions result in greater metastatic capacity; Whereas SCH772984 suppressed EMT transition of cancer cells, and upregulated senescence markers p15 and p16, malignancy-related genes MMP2, MMP3 and IL-6, and fibrosis markers α-SMA and Collagen Type I in PSCs. Also, its combination with an autophagy inhibitor, chloroquine, suppresses SCH772984-induced autophagy. Therefore, the combination therapy possibly leads to strong induction of cellular senescence in PSCs. In summary, dual treatment with ERK inhibitor and CQ inhibit cancer–stromal interaction and metastatic capacity

In the present study, we investigated p-ERK1/2 expression in PCCs and PSCs, and its functional impact on pancreatic cancer–stromal interaction. Our results showed that ERK1/2 activation is upregulated during PCC metastasis and PCC–PSC interaction. Inhibition of p-ERK1/2 in PCCs and PSCs induced remarkable cell viability repression and changes in mRNA expression. Delivery of ERK inhibitor repressed cancer–stromal interaction via a PSC-preferential behavior. We also found ERK inhibition to induce autophagy in PSCs, and this effect was suppressed by combining the ERK inhibitor with the autophagy inhibitor, CQ (Fig. 8).

Previous studies have focused mainly on the RAS-RAF-MEK-ERK pathway in cancer cells. However, p-ERK1/2 expression has been shown prognostic and metastatic implications in PDAC [40, 41]. Our result showed HMPDAC cells had high levels of activated ERK1/2, whereas inhibiting ERK1/2 suppressed EMT in these cells. Furthermore, activation of ERK1/2 also increased in PDAC cells after co-culture with PSCs. These findings indicate that activation of ERK1/2 promotes metastasis and cancer–stromal interaction in PDAC cells. We found that activation of ERK1/2 was frequently observed in stroma even compared with PCCs in the resected pancreatic cancer tissues. Moreover, cancer-associated PSCs were more sensitive to ERK inhibitor SCH772984 than cancer cells. Therefore, the present research focused on the activation of ERK1/2 in PSCs as well as PCCs. Cancer-associated PSCs are the major components of extensive desmoplasia, which contribute to PDAC progression and chemoresistance [42]. Recently, anti-stromal drugs were reported to inhibit PDAC progression via suppression of PSCs [43, 44]. These findings suggest that inhibiting PSCs could be the basis of an effective therapeutic strategy for PDAC.

Inhibition of ERK pathway increased sensitization to gemcitabine of PDAC cells and PDAC xenograft mouse model [43]. The present data demonstrated that ERK inhibitor improved chemosensitivity of gemcitabine of PDAC cells in the PSC-conditioned medium. However, our results suggest that inhibiting ERK1/2 is a two-edged sword that simultaneously induces autophagy and suppresses cell viability in PSCs. Autophagy in PDAC stroma is associated with accelerated cancer progression; high expression of LC3-II, an autophagy marker, in PDAC specimens is prognostic of poor survival [23]. Immunohistochemical analysis of mouse fibroblasts revealed increased co-localization of p-ERK1/2 with LC3-II and Autophagy-related proteins such as Atg5, Atg12 and Atg16 [45]. Cytoplasmic sequestration of ERK1/2 has been shown to promote autophagy in human ovarian cancer cells [46]. However, autophagy prevents cellular senescence [47]. Therefore, ERK1/2 inhibition did not induce senescence in PSCs, despite increased expression of p15 and p16. The protein p16 is a major player in cellular response to DNA damage, which leads to senescence [48] and/or apoptosis [49, 50]. Overexpression of p16 activates autophagy and cellular senescence in both human fibroblasts and breast cancer cells [51]. However, the mechanism of ERK inhibitor-induced autophagy is unclear. Although further investigation is needed, it may be initiated by p16-induced DNA damage, which upregulates p53; p53 then activates transcription of several autophagy-related genes including *ATG5* and *ATG7* [52]. As inhibition of autophagy induces cellular senescence in primary human fibroblasts [53]. we chose CQ as our combination drug, thus we obtained a satisfactory result.

Tumor organoid models are a new tool in biomedical research, and have been recently used to explore the effects of p-ERK1/2 inhibitors on several types of cancer, including ERK inhibitor SCH772984 in hepatocellular carcinoma [38] and bladder cancer [54]. SCH772984 also suppressed formation and viability of patient-derived pancreatic organoids [55]. In the present study, we performed in vivo xenograft experiment using patient-derived PDAC organoids, and found that ERK inhibitor treatment alone reduced the number of metastatic nodules and Ki67 expression in liver metastases. Compared to 2D-adherent cultured cells, pancreatic cancer organoids demonstrated greater p-ERK1/2 expression, which was consistent with the findings in original resected PDAC tumors, and suggests that organoid models can be used to investigate the therapeutic effects of ERK inhibitors, due to the reproducible p-ERK1/2 expression, which is consistent with that in resected samples.

## Conclusion

In summary, inhibition of p-ERK1/2 preferentially suppressed cancer–stromal interaction by decreasing viability of PSCs. However, ERK inhibitor also induced autophagy and may have prevented senescence of the activated PSCs. Our findings also indicate that combined inhibition of ERK1/2 and autophagy significantly decreased the number, volume and weight of liver metastases. Taken together, combination therapy to suppress ERK1/2 and autophagy is a potential treatment for pancreatic cancer.

## Additional files

Additional file 1: **Figure S1.** Characteristics of highly metastatic PDAC cells. (A) Suspended cell viability of cancer cells as determined by CellTiter-Glo luminescent cell viability assay. **P < 0.01. (B) Tumor weight but not volume was significantly increased in dual-treatment group. *P < 0.05. (C) SLMS/SUIT-2 ratio for protein kinases expression of significance. (D) Western blot of ERK1/2, p-ERK1/2, E-cadherin and vimentin levels in highly metastatic and parental PDAC cells. (PDF 262360 kb)

Additional file 2: **Figure S2.** Expression and prognosis of p-ERK1/2 in PDAC database. (A) ERK1 and (B) ERK2 expression was detected in both cancer and stromal cells in human pancreatic primary tumor. (C, D) Kaplan–Meier survival analysis of overall survival and disease-free survival of patients with pancreatic cancer by *ERK1* and *ERK2* mRNA expression. (PDF 262364 kb)

Additional file 3: **Figure S3.** Dual-treatment with SCH772984 and CQ decreased cell viability and induced PSC2 senescence. (A) Microphotograph of PSC2 cells after treatment with indicated agents. Scale bars = 100 μm. (B) Viability of PSCs after treatment with indicated agents. (C) qRT-PCR showed mRNA expression of *α-SMA*, Collagen Type I, *p15* and *p16*. *P < 0.05, **P < 0.01, ***P < 0.001. (D) β-galactosidase staining of PSC2 cells after treatment with indicated agents. Graphs show the quantification of β-gal-positive cells calculated from five fields. Scale bars =100 μm. *P < 0.05. (E) Migration and invasion assays were performed for 18 and 36 h, respectively. Graphs show numbers of cells calculated from five fields. Scale bars =100 μm. *P < 0.05. (F) Cell viability of PCCs after indicated agent treatment. Columns, mean fold changes of three experiments done in triplicate. (G) Western blot of indicated protein levels of PCCs after indicated treatment. (PDF 262364 kb)

Additional file 4: **Figure S4.** Pancreatic tumor organoid recapitulates p-ERK1/2 expression of PDAC in vitro and in vivo. (A) Western blot of ERK1/2 and p-ERK1/2 shows increasing p-ERK1/2 level in KPC cancer organoid compared with cancer cells. (B) Scheme of xenograft experiment. Mice were randomly divided into two groups and 5 × 10^4^ cancer cells or organoids were intrasplenic implanted with 5 × 10^4^ PSCs. After 2 weeks, liver metastases were evaluated. (C) H&E staining and immunohistochemical staining show metastatic nodules (D), and expression of α-SMA (E) and p-ERK1/2 (F). (G, H) Combination of indicated dose of SCH772984 improved chemosensitivity of gemcitabine of AsPC-1 and SUIT-2 cells in the PSC-conditioned medium. Columns, mean fold changes of three experiments done in triplicate. (PDF 262364 kb)

Additional file 5: **Table S1.** Primers used for quantitative RT-PCR. (DOCX 56 kb)

Additional file 6: **Table S2.** Clinicopathological features of PADC samples used for p-ERK1/2 immunohistochemistry. (XLSX 11 kb)

## Abbreviations

CQ: chloroquine
EMT: epithelial–mesenchymal transition
ERK: Extracellular signal-regulated kinases
HM: highly metastatic
HMPCC: highly metastatic pancreatic cancer cell
HPDE: Human pancreatic ductal epithelial
IC50: 50% inhibitory concentration
KPC: LSL-Kras G12D/+
LSL: Trp53R172H/+
MAPK: Mitogen-activated protein kinases
OS: overall survival
PCC: pancreatic cancer cell
PDAC: Pancreatic ductal adenocarcinoma
Pdx: 1-Cre
PSC: pancreatic stellate cell
