# Supplementary material for: Inhibition of ERK1/2 in cancer-associated pancreatic stellate cells suppresses cancer–stromal interaction and metastasis
Source: J Exp Clin Cancer Res. 2019 May 27;38:221. doi: 10.1186/s13046-019-1226-8 (PMC6537367; doi:10.1186/s13046-019-1226-8)
Supplement: Supplementary file 5 — Table S1. Primers used for quantitative RT-PCR. (DOCX 56 kb) [file 13046_2019_1226_MOESM5_ESM.docx]

Table S1. Primers used for quantitative RT-PCR.

| Primer | Forward sequence 5’-3’ | Reverse sequence 5’-3’ |
| --- | --- | --- |
| αSMA | GACAATGGCTCTGGGCTCTGTAA | CTGTGCTTCGTCACCCACGTA |
| Fibronectin | ACAGAACTATGATGCCGACCAGAAG | ACTGATCTCCAATGCGGTACATGA |
| Collagen Type I | TCTAGACATGTTCAGCTTTGTGGAC | TCTGTACGCAGGTGATTGGTG |
| Collagen Type VI | ACCTACACCGACTGCGCTAT | TCGGTCACCACAATCAGGTA |
| MMP2 | TGACATCAAGGGCATTCAGGAG | TCTGAGCGATGCCATCAAATACA |
| mmp3 | ATTCCATGGAGCCAGGCTTTC | CATTTGGGTCAAACTCCAACTGTG |
| mmp14 | GTCTCCCAGAGGGTCATTCA | GGTAGCCCGGTTCTACCTTC |
| IL-6 | AAGCCAGAGCTGTGCAGATGAGTA | TGTCCTGCAGCCACTGGTTC |
| p15 | GCGGATTTCCAGGGATATTT | CACCAGGTCCAGTCAAGGAT |
| p16 | GAGCAGCATGGAGCCTTC | GGCCTCCGACCGTAACTATT |
