# Supplementary material for: Inhibition of ERK1/2 in cancer-associated pancreatic stellate cells suppresses cancer–stromal interaction and metastasis
Source: J Exp Clin Cancer Res. 2019 May 27;38:221. doi: 10.1186/s13046-019-1226-8 (PMC6537367; doi:10.1186/s13046-019-1226-8)

Fig. S1

A

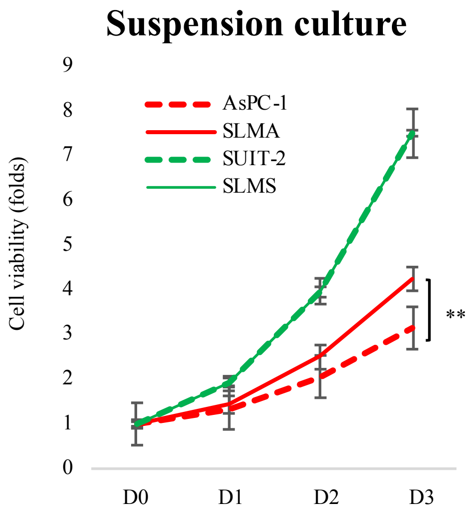

D

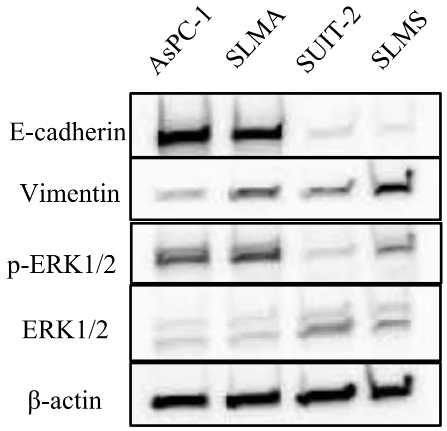

B

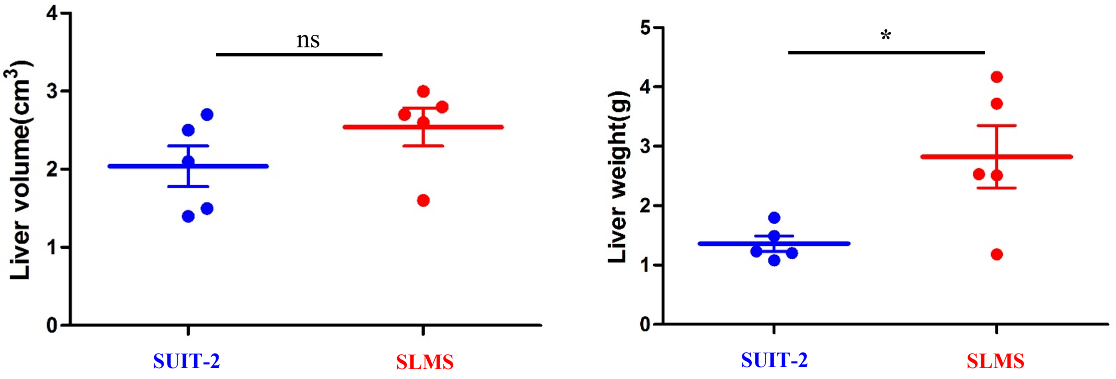

C

SLMS/ SUIT-2 ratio

**ERK1/2 (T202/Y204, T185/ Y187)**<br>2.67	**GSK-3α/β (S21/S9)**<br>1.34	**AMPLa1(T183)**<br>1.33	**Akt1/2/3 (S473)**<br>2.32
**p53 (S46)**<br>1.87	**AMPKα2 (T172)**<br>0.65	**β-Catenin**<br>0.65	**Src (Y419)**<br>1.45
**p70 S6 Kinase (Y419)**<br>0.73	**Fyn(Y420)**<br>1.83	**Yes (Y426)**<br>1.46	**STAT6 (Y641)**<br>0.83
**Chk-2 (T68)**<br>1.48	**FAK (Y397)**<br>1.22	**WNK1 (T60)**<br>2.37	**HSP60**<br>0.8

E

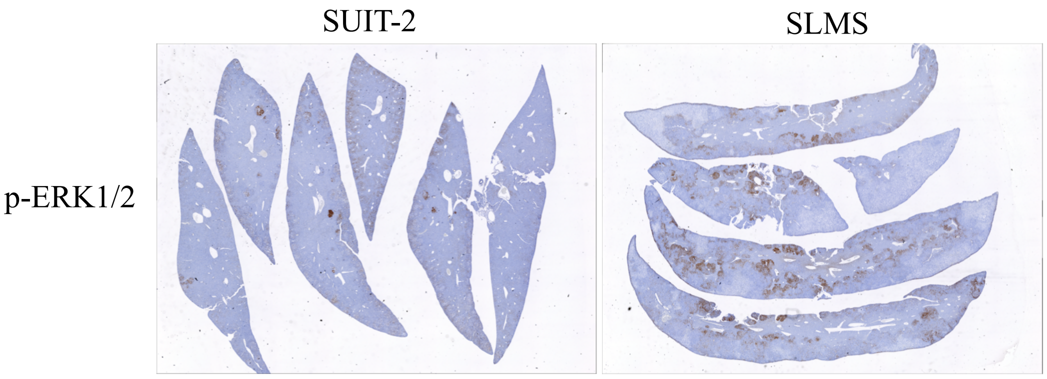

Supplement: Supplementary file 1 — Figure S1. Characteristics of highly metastatic PDAC cells. (A) Suspended cell viability of cancer cells as determined by CellTiter-Glo luminescent cell viability assay. **P < 0.01. (B) Tumor weight but not volume was significantly increased in dual-treatment group. *P < 0.05. (C) SLMS/SUIT-2 ratio for protein kinases expression of significance. (D) Western blot of ERK1/2, p-ERK1/2, E-cadherin and vimentin levels in highly metastatic and parental PDAC cells. (PDF 262360 kb) [file 13046_2019_1226_MOESM1_ESM.pdf]
