# Supplementary material for: Inhibition of ERK1/2 in cancer-associated pancreatic stellate cells suppresses cancer–stromal interaction and metastasis
Source: J Exp Clin Cancer Res. 2019 May 27;38:221. doi: 10.1186/s13046-019-1226-8 (PMC6537367; doi:10.1186/s13046-019-1226-8)
Supplement: Supplementary file 2 — Figure S2. Expression and prognosis of p-ERK1/2 in PDAC database. (A) ERK1 and (B) ERK2 expression was detected in both cancer and stromal cells in human pancreatic primary tumor. (C, D) Kaplan–Meier survival analysis of overall survival and disease-free survival of patients with pancreatic cancer by ERK1 and ERK2 mRNA expression. (PDF 262364 kb) [file 13046_2019_1226_MOESM2_ESM.pdf]

Fig. S2

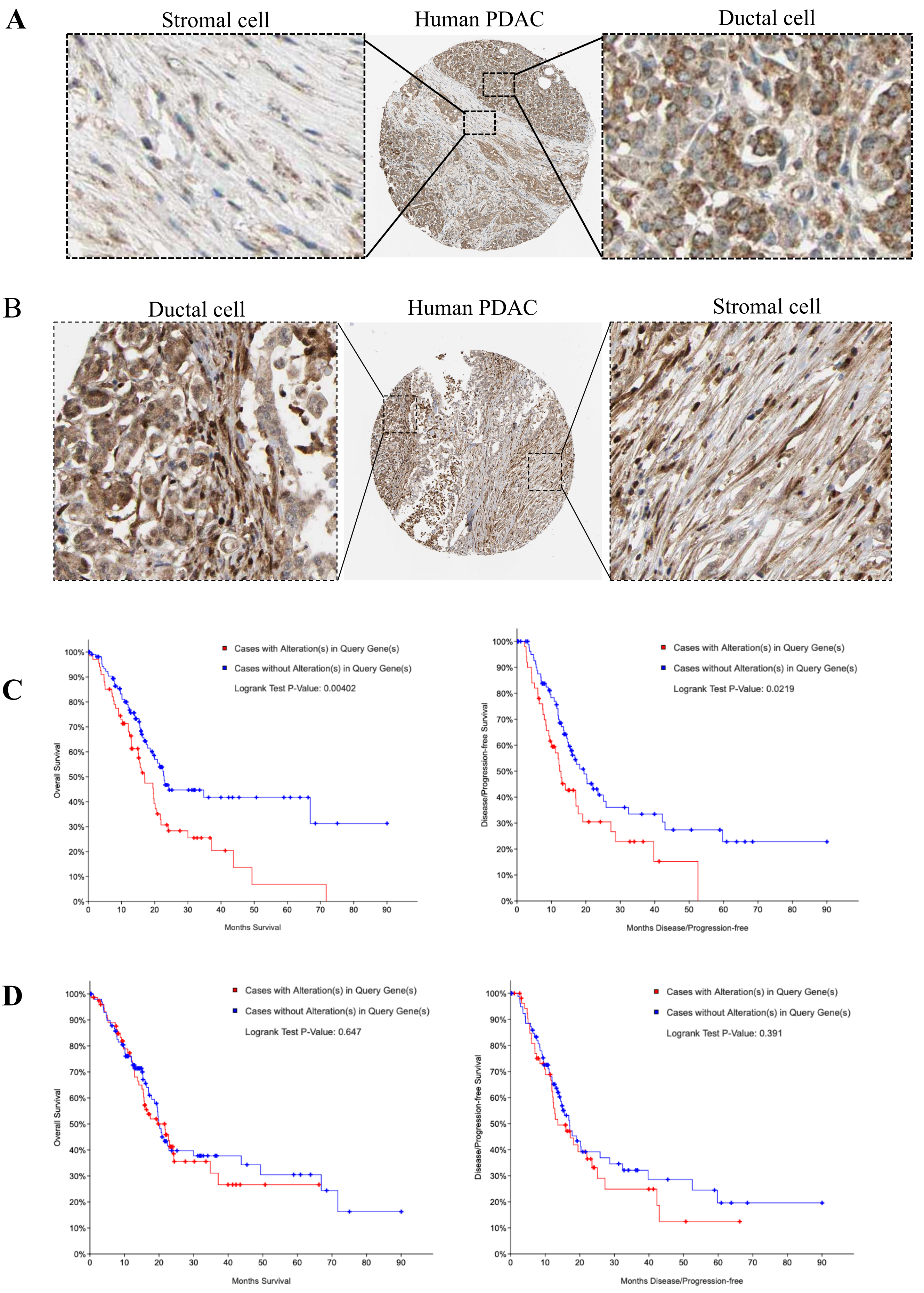
A
Stromal cell
Human PDAC
Ductal cell
B
Ductal cell
Human PDAC
Stromal cell
C
Cases with Alteration(s) in Query Gene(s)
Cases without Alteration(s) in Query Gene(s)
Logrank Test P-Value: 0.00402
Overall Survival
Months Survival
Cases with Alteration(s) in Query Gene(s)
Cases without Alteration(s) in Query Gene(s)
Logrank Test P-Value: 0.0219
Disease/Progression-free Survival
Months Disease/Progression-free
D
Cases with Alteration(s) in Query Gene(s)
Cases without Alteration(s) in Query Gene(s)
Logrank Test P-Value: 0.647
Overall Survival
Months Survival
Cases with Alteration(s) in Query Gene(s)
Cases without Alteration(s) in Query Gene(s)
Logrank Test P-Value: 0.391
Disease/Progression-free Survival
Months Disease/Progression-free
