# Supplementary material for: Inhibition of ERK1/2 in cancer-associated pancreatic stellate cells suppresses cancer–stromal interaction and metastasis
Source: J Exp Clin Cancer Res. 2019 May 27;38:221. doi: 10.1186/s13046-019-1226-8 (PMC6537367; doi:10.1186/s13046-019-1226-8)
Supplement: Supplementary file 3 — Figure S3. Dual-treatment with SCH772984 and CQ decreased cell viability and induced PSC2 senescence. (A) Microphotograph of PSC2 cells after treatment with indicated agents. Scale bars = 100 μm. (B) Viability of PSCs after treatment with indicated agents. (C) qRT-PCR showed mRNA expression of α-SMA, Collagen Type I, p15 and p16. *P < 0.05, **P < 0.01, ***P < 0.001. (D) β-galactosidase staining of PSC2 cells after treatment with indicated agents. Graphs show the quantification of β-gal-positive cells calculated from five fields. Scale bars =100 μm. *P < 0.05. (E) Migration and invasion assays were performed for 18 and 36 h, respectively. Graphs show numbers of cells calculated from five fields. Scale bars =100 μm. *P < 0.05. (F) Cell viability of PCCs after indicated agent treatment. Columns, mean fold changes of three experiments done in triplicate. (G) Western blot of indicated protein levels of PCCs after indicated treatment. (PDF 262364 kb) [file 13046_2019_1226_MOESM3_ESM.pdf]

Fig. S3

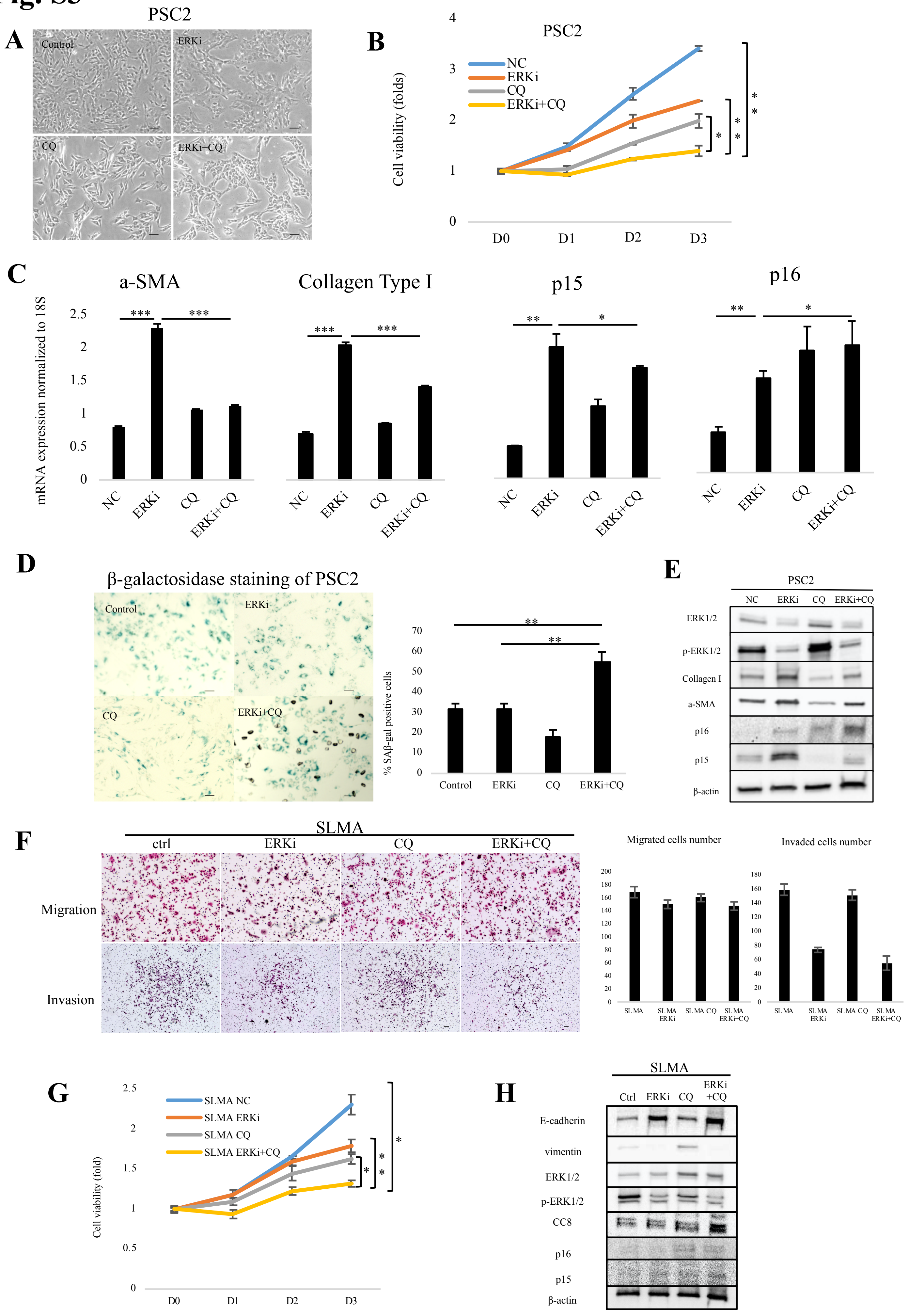

PSC2
A
Control
ERKi
CQ
ERKi+CQ
B
PSC2
NC
ERKi
CQ
ERKi+CQ
Cell viability (folds)
D0
D1
D2
D3
C
a-SMA
Collagen Type I
p15
p16
mRNA expression normalized to 18S
NC
ERKi
CQ
ERKi+CQ
D
β-galactosidase staining of PSC2
Control
ERKi
CQ
ERKi+CQ
% SAβ-gal positive cells
E
PSC2
NC
ERKi
CQ
ERKi+CQ
ERK1/2
p-ERK1/2
Collagen I
a-SMA
p16
p15
β-actin
F
SLMA
ctrl
ERKi
CQ
ERKi+CQ
Migration
Invasion
Migrated cells number
Invaded cells number
SLMA
SLMA ERKi
SLMA CQ
SLMA ERKi+CQ
G
SLMA NC
SLMA ERKi
SLMA CQ
SLMA ERKi+CQ
Cell viability (fold)
D0
D1
D2
D3
H
SLMA
Ctrl
ERKi
CQ
ERKi +CQ
E-cadherin
vimentin
ERK1/2
p-ERK1/2
CC8
p16
p15
β-actin
