# Supplementary material for: Inhibition of ERK1/2 in cancer-associated pancreatic stellate cells suppresses cancer–stromal interaction and metastasis
Source: J Exp Clin Cancer Res. 2019 May 27;38:221. doi: 10.1186/s13046-019-1226-8 (PMC6537367; doi:10.1186/s13046-019-1226-8)
Supplement: Supplementary file 4 — Figure S4. Pancreatic tumor organoid recapitulates p-ERK1/2 expression of PDAC in vitro and in vivo. (A) Western blot of ERK1/2 and p-ERK1/2 shows increasing p-ERK1/2 level in KPC cancer organoid compared with cancer cells. (B) Scheme of xenograft experiment. Mice were randomly divided into two groups and 5 × 10^4^ cancer cells or organoids were intrasplenic implanted with 5 × 10^4^ PSCs. After 2 weeks, liver metastases were evaluated. (C) H&E staining and immunohistochemical staining show metastatic nodules (D), and expression of α-SMA (E) and p-ERK1/2 (F). (G, H) Combination of indicated dose of SCH772984 improved chemosensitivity of gemcitabine of AsPC-1 and SUIT-2 cells in the PSC-conditioned medium. Columns, mean fold changes of three experiments done in triplicate. (PDF 262364 kb) [file 13046_2019_1226_MOESM4_ESM.pdf]

Figure S4

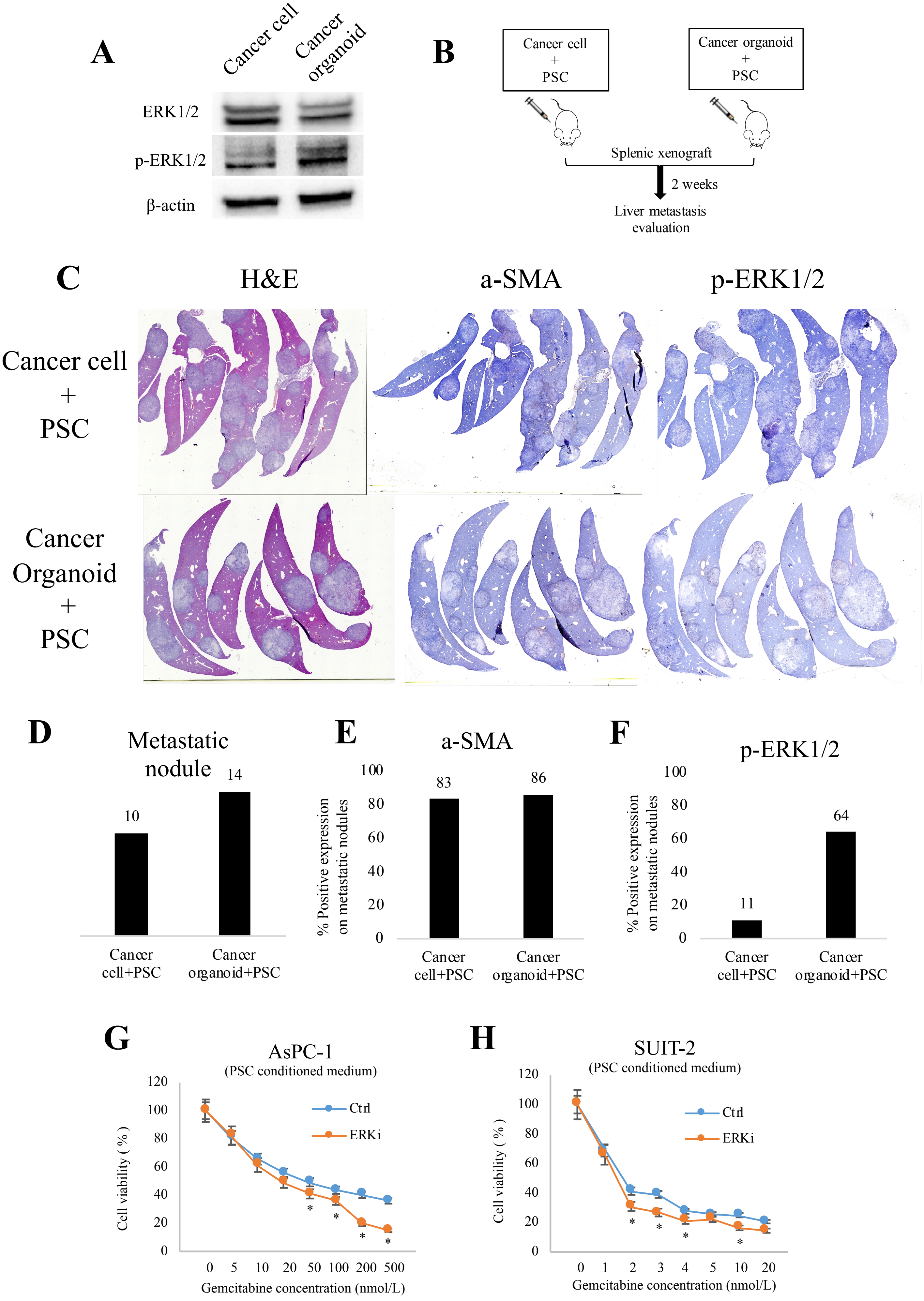

A
Cancer cell
Cancer organoid
ERK1/2
p-ERK1/2
β-actin
B
Cancer cell + PSC
Cancer organoid + PSC
Splenic xenograft
2 weeks
Liver metastasis evaluation
C
H&E
a-SMA
p-ERK1/2
Cancer cell + PSC
Cancer Organoid + PSC
D
Metastatic nodule
10
14
Cancer cell+PSC
Cancer organoid+PSC
E
a-SMA
% Positive expression on metastatic nodules
100
80
60
40
20
0
83
86
Cancer cell+PSC
Cancer organoid+PSC
F
p-ERK1/2
% Positive expression on metastatic nodules
100
80
60
40
20
0
11
64
Cancer cell+PSC
Cancer organoid+PSC
G
AsPC-1
(PSC conditioned medium)
Cell viability (%)
120
100
80
60
40
20
0
Ctrl
ERKi
*
*
*
*
0 5 10 20 50 100 200 500
Gemcitabine concentration (nmol/L)
H
SUIT-2
(PSC conditioned medium)
Cell viability (%)
120
100
80
60
40
20
0
Ctrl
ERKi
*
*
*
*
0 1 2 3 4 5 10 20
Gemcitabine concentration (nmol/L)
